# Living with Dementia During the COVID-19 Pandemic: A Nationwide Survey Informing the American Experience

**DOI:** 10.3233/ADR-220085

**Published:** 2022-11-24

**Authors:** Paul Arthur, Chih-Ying Li, The Southern Indiana Dementia Workgroup

**Affiliations:** a Department of Occupational Therapy, University of Southern Indiana, Evansville, IN, USA; b Departmentof Occupational Therapy, University of Texas Medical Branch, Galveston, TX, USA; c Evansville, IN, USA

**Keywords:** Alzheimer’s disease, behaviors, caregiving, COVID-19, dementia, neuropsychiatric symptoms, pandemic, public health emergency

## Abstract

Persons living with dementia and their caregivers are among society’s most vulnerable, a condition exacerbated by the COVID-19 pandemic. This national survey was conducted with dementia caregivers in the US. Primary outcomes targeted pandemic-related changes in cognitive, behavioral, and motors systems. 113 dementia caregivers from 30 US states completed the survey. The impact of the COVID-19 pandemic on persons living with dementia and their caregivers is substantial in comparison to society at large. A marked public health and preventative role signals opportunity for practitioners to fill the void and prepare for future public health emergencies.

## INTRODUCTION

More than 6 million Americans have Alzheimer’s disease or related dementias (ADRD) and approximately 85% live in the community [1, 2]. With prevalence in the United States (US) expected to near 14 million by 2050, ADRD has been identified as a rising epidemic with urgent need for intervention [1, 3]. Alzheimer’s disease is characterized as a progressive and irreversible deterioration in cognitive ability negatively impacting one’s ability to live and function independently [4]. Decreased cognition paired with neuropsychiatric symptoms and physical dependence often leads to challenging situations for caregivers who are experiencing concurring lifestyle/role changes of their own.

Persons living with dementia (PLWD) regularly rely on informal/unpaid caregivers for daily support and safety and such support comes at an extraordinary cost to the caregiver. In the US, there are approximately 15.7 million informal or family caregivers of PLWD that furlough their lives to provide such support [5]. Reports suggest that as many as 80% of caregivers of PLWD experience high levels of stress [6] and nearly 40% experience significant depressive episodes [7]. Female caregivers, those going through multiple life events/changes, and caregivers lacking significant social networks have the highest risk for CG-related distress [8] which is often described as the result of a mix of circumstances, experiences, responses, and resources [9].

On January 20, 2020, the Centers for Disease Control and Prevention (CDC) reported the first laboratory-confirmed COVID-19 in the US; by March 11th the World Health Organization declared a global pandemic and US states began to shut down to prevent spread [10]. As of November 2022, COVID variants remain within the US and aging/vulnerable adults are encouraged to continue preventative measures to protect themselves [11].

People with dementia and their caregivers have historically been among society’s most vulnerable. Rodeheaver & Datan [12] surmised “As probably one of the most excluded groups in society, people with dementia experience the double jeopardy of being (aging) people with a cognitive impairment” (p.1). COVID-related restrictions to manage the pandemic in the US have amplified this inequity. For more than 2 years, PLWD and their caregivers have been asked to isolate from support systems, change routines, and decrease service use [13, 14]. Prolonged isolation can further feelings of loneliness, behavioral disruptions, and acute events, all while medical care is less accessible [14]. PLWD are less likely to adhere to precautions and have a markedly greater risk of experiencing a COVID infection than peers [15]. Mortality in infection is 40% higher than in peers as well, with 20% of PLWD with COVID living less than 6 months [16].

Despite the many negative outcomes associated with the COVID-19 pandemic, some have reported positive aspects from the caregiving experience in this time. As reported by Tulloch, McCaul, & Scott [17], mandated isolation allowed for a strengthened connection for the person living with dementia and their caregiver and a renewed/greater sense of meaning in the caring experience. Though present research suggests more obstacles than opportunities remain.

## METHODS

### Sample and settings

This study was approved by the Institutional Review Board (IRB # 1901305-1). The cross-sectional national survey was released in the Spring of 2022 and utilized a paid social media clickable advertising algorithm targeting individuals who identified with the dementia caregiving community. A total of $500 (USD) was budgeted and spent for recruitment. Inclusion criteria were persons living within the US, 18 years or older, and that cared for someone living with dementia during the COVID-19 pandemic.

### Measures

Dementia stage was assessed with the Clinical Dementia Rating [18] and the primary and secondary outcomes from a semi-structured questionnaire created by Rainero et al. [19]. The primary outcomes were to identify changes in cognitive, behavioral, and motor symptoms in PLWD. Secondary outcomes were to identify effects on caregivers’ well-being.

### Statistical analysis

We completed statistical analysis using SAS software, version 9.4M7. We completed descriptive analysis of demographic and clinical baseline data. We then completed univariable and multivariable logistic regression of the dependent variables on the collected independent variables using mixed effects logistic regression. Regressors with significant p values in univariable logistic regression were included in multivariable regression. Bonferroni correction was applied to all the *p*-values of multivariable analysis, considering all outcomes together. Statistical significance was set at *p* < 0.05.

## RESULTS

### Demographics

Demographics are detailed in Table 1. The mean age of persons living with dementia was 80 years (SD = 9.4), the majority were female (51%) with a disease duration of nearly 6 years (SD = 4.4) at the time of data collection. The mean age of caregivers was nearly 64 (SD = 9.9), predominantly female (95%), and were primarily “spouses”.

**Table 1:** Clinical information

	Total (*n* = 113)
Disease duration (y, mean, SD)	5.94 (4.43)
Dementia type (freq, %)
Alzheimer’s disease	43 (45.7)
Lewy body	6 (6.4)
Vascular	22 (23.4)
Mixed	2 (2.1)
Early-onset	2 (2.1)
Frontotemporal	3 (3.2)
Unknown	16 (17)
Clinical Dementia Rating Stage (%)
0.5	3
1	44
2	42
3	24
Symptom change
Changes in cognition (Yes, %)	91 (81.3)
Changes in neuropsychiatric symptoms (Yes, %)	88 (77.9)
New neuropsychiatric symptoms (Yes, %)	82 (72.6)
Number of new neuropsychiatric symptoms (mean, SD)	2.04 (1.74)
Change in motor symptoms (Yes, %)	89 (80.2)
Need to change medication due to symptoms
Yes (%)	68 (68.2)
Prior physical ability (yes, %)
Leave home independently	43 (37)
Leave home accompanied	67 (57.8)
Cannot leave home	6 (5.2)
Disease progress faster during pandemic
Yes (%)	65 (57.5)
Person living with dementia aware of pandemic
Yes (%)	51 (45.1)
Partially (%)	44 (38.9
No (%)	18 (15.9)

### Clinical information

Clinical information is detailed in Table 2. Perceived cognitive changes during the pandemic were reported in 80% of the PLWD. Perceived changes in behavioral symptoms were reported in 78% of the sample, with an average of 2.04 new symptoms appearing during the public health emergency. Perceived motor symptom changes were apparent in 81% of the sample. Healthcare service availability is reported in Table 3, where dementia services were reportedly reduced in more than 70% of cases, hospitalization unavailable in nearly 30%, and a lack of urgent service availability for dementia-specific needs including cognition and behavioral symptoms, as well as significantly reduced availability of semi-residential/residential services (e.g., adult day programs).

**Table 2:** Demographic information

	Total (*n* = 113)
Persons living with dementia
Age (y, mean, SD)	80.27 (9.36)
Gender (female, %)	51.3
Regional distribution
West (%)	23
Midwest (%)	14
North-East (%)	42
South (%)	24
Caregivers
Age (y, mean, SD)	63.7 (9.96)
Sex (female, %)	107 (94.7)
Living together (yes, %)	72 (64.3)
*Relationship Type*
Spouse (%)	42.5
Child (%)	33.6
Friend (%)	0.9
Sibling (%)	2.7
Other (%)	20.4

**Table 3:** Service availability

	Total (*n* = 110)
Hospitalization availability
Yes (%)	71 (64)
Dementia services availability
Yes (%)	32 (29.3)
Partially (%)	35 (32.1)
No (%)	42 (38.5)
Urgent dementia services for neuropsychiatric
symptoms or cognition available
Yes (%)	52 (48.6)
Semi-residential/residential services available
Yes (%)	19 (18.1)

Caregivers reported significant life disruption with the onset of the COVID-19 pandemic. Nearly 60% reported receiving no outside help during the shutdown; 65% endorsed feelings of abandonment, 60% being overwhelmed during the COVID-19 emergency. More than 60% endorsed significant life change, 35% increased conflicts with the PLWD, 65% concern about COVID-19 consequences for the PLWD, and 27% problems receiving consistent medical care. In logistic regression, PLWD prior-pandemic level of physical function (as reported as ability to leave the house with/without assistance) served as a protective factor for symptom change (odds ratio 0.20 [95% CI 0.00–0.29]).

## DISCUSSION

The purpose of this study was to increase understanding of the influence of the COVID-19 pandemic within the dementia community in the US. Our findings coincide with work by Canevelli et al. [20] that has suggested that the pandemic had profound impact on persons living with dementia and those who care for them. Despite the progressive nature of the disease, evidence increasingly suggests [21] a hastened cognitive decline during this period, as did our results with a mean of more than two new symptoms occurring within the relatively short public health emergency period and generally surpassing historical prognosis found in longitudinal studies [22]. It is also recognized that within a variable, progressive disease and in an observational study design, there may be additional variables that account for this numeric change beyond the public health emergency.

More nuanced information is coming to light regarding the increase in neuropsychiatric symptoms, in part, secondary to measures of physical and social isolation [23]. Pandemic-related isolation also seems to have hastened motor skill decline nationally [24], which is reflected in our US-based sample. As reported by Rainero et al. [19], we also found a signal of a protective mechanism in the pre-pandemic physical ability of the person living with dementia (as reported by the ability to leave the house with/without assistance) in preventing significant increases of neuropsychiatric symptoms, suggesting that there is nearly an 80% decrease in the odds of increased neuropsychiatric symptoms with heightened reported physical ability. The reported challenges of the healthcare system at large to provide hospitalization services, urgent and routine dementia services, and residential services signals a significant need to care for this growing community.

Caregiver compromise throughout the public health emergency was also notable and amplified. As shared in work by Greenberg et al. [13], isolation leads to higher-hour care which then leads to increased levels of housework, and increased challenges in coordinating care within and outside of the home. This was reflected in our results, signaling barriers in access and receipt of care. Caregivers are then dually disadvantaged due to the need to isolate themselves and the person living with dementia to lessen the risk of transmission and mortality, while paying a high price in their own social, mental, and physical well-being.

### Conclusion

While many have looked and planned beyond the COVID-19 pandemic, the impact on persons living with dementia and their caregivers was substantial in comparison to society at large. A marked public health and preventative role signals a timely opportunity for practitioners and scientists alike to fill the void and prepare for future public health emergencies and isolation periods for societies most vulnerable. There too are industrial and organizational roles to further support and prepare adult care service and resource centers for future public health emergencies, along with those providing resilience and respite services for caregivers of persons with dementia. For many of societies vulnerable, the pandemic is not over, nor is our work.

## References

[1] Alzheimer’s Association (2022) 2022 Alzheimer’s disease facts and figures. Alzheimers Dement 18, 700–789. 35289055 10.1002/alz.12638

[2] Chi W , Graf E , Hughes L , Hastie J , Khatutsky G , Shuman S , Lamont H (2019) Community-dwelling older adults with dementia and their caregivers: Key indicators from the national health and aging trends study. US Department of Health and Human Services, Office of the Assistant Secretary for Planning and Evaluation, Office of Disability, Aging and Long-Term Care Policy.

[3] Hickman RA , Faustin A , Wisniewski T (2016) Alzheimer disease and its growing epidemic: Risk factors, biomarkers, and the urgent need for therapeutics. Neurol Clin 34, 941–953. 27720002 10.1016/j.ncl.2016.06.009 PMC5116320

[4] Prince M , Bryce R , Albanese E , Wimo A , Ribeiro W , Ferri CP (2013) The global prevalence of dementia: A systematic review and metaanalysis. Alzheimers Dement 9, 63–75. 23305823 10.1016/j.jalz.2012.11.007

[5] Minkemeyer V , Wellman C , Goebel L (2016) The impact of Alzheimer’s disease in an aging rural population. W V Med J 112, 90–94. 27301161

[6] Schulz R , O’Brien A , Czaja S , Ory M , Norris R , Martire LM , Belle SH , Burgio L , Gitlin L , Coon D , Burns R , Gallagher-Thompson D , Stevens A (2002) Dementia caregiver intervention research: In search of clinical significance. Gerontologist 42, 589–602. 12351794 10.1093/geront/42.5.589 PMC2579772

[7] Arthur P , Gitlin LN , Kairalla J , Mann WC (2018) Relationship between the number of behavioral symptoms in dementia and caregiver distress: What is the tipping point? Int Psychogeriatr 30, 1099–1107. 29143722 10.1017/S104161021700237X PMC7103581

[8] Kim H , Chang M , Rose K , Kim S (2012) Predictors of caregiver burden in caregivers of individuals with dementia. J Adv Nurs 68, 846–855. 21793872 10.1111/j.1365-2648.2011.05787.x

[9] Pearlin LI , Mullan JT , Semple SJ , Skaff MM (1990) Caregiving and the stress process: An overview of concepts and their measures. Gerontologist 30, 583–594. 2276631 10.1093/geront/30.5.583

[10] Centers for Disease Control and Prevention (2022) CDC Museum COVID-19 Timeline. https://www.cdc.gov/museum/timeline/covid19.html, Accessed September 1, 2022.

[11] Centers for Disease Control and Prevention (2021) COVID-19 risks and vaccine information for older adults: Older unvaccinated adults are more likely to be hospitalized or die from COVID-19.https://www.cdc.gov/aging/covid19/covid19-older-adults.html#: :text=What%20you%20need%20to%20know,years%20of%20age%20and%20older, Accessed September 1, 2022.

[12] Rodeheaver D , Datan N (1988) The challenge of double jeopardy: Toward a mental health agenda for aging women. Am Psychol 43, 648–654. 3052197 10.1037//0003-066x.43.8.648

[13] Greenberg NE , Wallick A , Brown LM (2020) Impact of COVID-19 pandemic restrictions on community-dwelling caregivers and persons with dementia. Psychol Trauma 12(S1), S220–S221. 32584105 10.1037/tra0000793

[14] Wong BPS , Kwok TCY , Chui KCM , Cheng TST , Ho FKY , Woo J (2021) The impact of dementia daycare service cessation due to COVID-19 pandemic. Int J Geriatr Psychiatry 37, 10.1002/gps.5621. PMC8646460 34490680

[15] Wang L , Shen Y , Li M , Chuang H , Ye Y , Zhao H , Wang H (2020) Clinical manifestations and evidence of neurological involvement in 2019 novel coronavirus SARS-CoV-2: A systematic review and meta-analysis. J Neurol 267, 2777–2789. 32529575 10.1007/s00415-020-09974-2 PMC7288253

[16] Bianchetti A , Rozzini R , Guerini F , Boffelli S , Ranieri P , Minelli G , Bianchetti L , Trabucchi M (2020) Clinical presentation of COVID19 in dementia patients. J Nutr Health Aging 24, 560–562. 32510106 10.1007/s12603-020-1389-1 PMC7227170

[17] Tulloch K , McCaul T , Scott TL (2022) Positive aspects of dementia caregiving during the COVID-19 pandemic. Clin Gerontol 45, 86–96. 34080958 10.1080/07317115.2021.1929630

[18] Morris JC , Ernesto C , Schafer K , Coats M , Leon S , Sano M (1997) Clinical dementia rating training and reliability in multicentre studies: The Alzheimer’s Disease Cooperative Study experience. Neurology 48, 1508–1510. 9191756 10.1212/wnl.48.6.1508

[19] Rainero I , Bruni AC , Marra C , Cagnin A , Bonanni L , Cupidi C , Laganà V , Rubino E , Vacca A , Di Lorenzo R , Provero P , Isella V , Vanacore N , Agosta F , Appollonio I , Caffarra P , Bussè C , Sambati R , Quaranta D , Guglielmi V , Logroscino G , Filippi M , Tedeschi G , Ferrarese C ; SINdem COVID-19 Study Group (2021) The impact ofCOVID-19 quarantine on patients with dementia and family caregivers:A nation-wide survey. Front Aging Neurosci 12, 507. 10.3389/fnagi.2020.625781 PMC7849158 33536898

[20] Canevelli M , Valletta M , Toccaceli Blasi M , Remoli G , Sarti G , Nuti F , Sciancalepore F , Ruberti E , Cesari M , Bruno G (2020) Facing dementia during the COVID-19 Outbreak. J Am Geriatr Soc 68, 1673–1676. 32516441 10.1111/jgs.16644 PMC7300919

[21] Tondo G , Sarasso B , Serra P , Tesser F , Comi C (2021) The Impact of the COVID-19 pandemic on the cognition of people with dementia. Int J Environ Res Public Health 18, 4285. 33919491 10.3390/ijerph18084285 PMC8073614

[22] Tschanz JT , Corcoran CD , Schwartz S , Treiber K , Green RC , Norton MC , Mielke MM , Piercy K , Steinberg M , Rabins PV , Leoutsakos JM , Welsh-Bohmer KA , Breitner JC , Lyketsos CG (2011) Progression of cognitive, functional, and neuropsychiatric symptom domains in a population cohort with Alzheimer dementia: The Cache County Dementia Progression study. Am J Geriatr Psychiatry 19, 532–542. 21606896 10.1097/JGP.0b013e3181faec23 PMC3101372

[23] Numbers K , Brodaty H (2021) The effects of the COVID-19 pandemic on people with dementia. Nat Rev Neurol 17, 69–70. 33408384 10.1038/s41582-020-00450-z PMC7786184

[24] Di Lorito C , Bosco A , Goldberg SE , Nair R , O’Brien R , Howe L , van der Wardt V , Pollock K , Booth V , Logan P , Godfrey M , Dunlop M , Horne J , Harwood RH (2020) Protocol for the process evaluation of the promoting activity, independence and stability in early dementia (PrAISED), following changes required by the COVID-19 pandemic. BMJ Open 10, e039305. 10.1136/bmjopen-2020-039305 PMC7453764 32859666

